# Diagnostic Value of Clinical and Optical Coherence Tomography Findings in Primary Vitreoretinal Lymphoma: A Retrospective Cohort Study

**DOI:** 10.3390/jcm15187193

**Published:** 2026-09-16

**Authors:** Mihai-Luca Cioboată, Ioana Tofolean, Suher Abduraman, Radu Burcea, Maria-Alexandra Chiotan-Călin, Radu-Alexandru Chiotan, Dana-Margareta-Cornelia Dăscălescu, Miruna Cioboată, Ali Rıza Cenk Çelebi, Florian Baltă

**Affiliations:** 1 Department of Ophthalmology, Carol Davila University of Medicine & Pharmacy, 020021 Bucharest, Romania; mihai-luca.cioboata@drd.umfcd.ro (M.-L.C.); radustefan505@yahoo.com (R.B.); maria-alexandra.chiotan@drd.umfcd.ro (M.-A.C.-C.); radu.chiotan@umfcd.ro (R.-A.C.); dana.dascalescu@umfcd.ro (D.-M.-C.D.); miruna.cioboata@umfcd.ro (M.C.); florian.balta@umfcd.ro (F.B.); 2 Department of Ophthalmology, Clinical Institute of Ophthalmological Emergencies “Prof. Dr. Mircea Olteanu”, 010464 Bucharest, Romania; abduramansuher@yahoo.com; 3 Retina Clinic, 014142 Bucharest, Romania; 4 National Institute of Pneumophthisiology “Marius Nasta”, 050159 Bucharest, Romania; 5 Department of Ophthalmology, Istanbul Medipol University, 34196 Istanbul, Türkiye

**Keywords:** primary vitreoretinal lymphoma, optical coherence tomography, intraocular lymphoma, visual acuity, vitreous biopsy, masquerade syndrome, diagnostic imaging

## Abstract

**Objectives:** Primary vitreoretinal lymphoma (PVRL) is a rare intraocular malignancy that can frequently masquerade as chronic uveitis, leading to substantial diagnostic delay. This study aimed to evaluate the diagnostic contribution of clinical examination and optical coherence tomography (OCT) in PVRL, to quantify the relationship between structural OCT findings and visual acuity (VA), and to identify factors associated with the yield of diagnostic vitrectomy. **Methods:** We retrospectively analyzed 17 patients with cytologically confirmed PVRL evaluated at two ophthalmology centers in Bucharest, Romania (2018–2023). Demographic, clinical, and OCT data were collected. An exploratory five-item OCT score (retinal infiltrates, sub-retinal pigment epithelium [sub-RPE] lesions, outer retinal hyperreflectivity, outer retinal disorganization, and foveal involvement) was constructed. Associations with VA (logMAR) were assessed using Spearman correlation, Mann–Whitney U, and Fisher’s exact tests. **Results:** Mean age was 66.1 ± 9.6 years (76.5% female); 76.5% had bilateral involvement at diagnosis, rising to 88.2% during follow-up. Median symptom duration before diagnosis was 250 days. The OCT score correlated strongly with VA (ρ = 0.772, *p* < 0.001). Outer retinal hyperreflectivity, sub-RPE lesions, retinal infiltrates, and outer retinal disorganization were each associated with significantly worse VA (all *p* < 0.05), whereas foveal involvement and subretinal fluid were not. A first vitrectomy was diagnostic in 82.4% of cases; prior corticosteroid exposure was significantly associated with an inconclusive initial cytology (Fisher’s exact *p* = 0.015). **Conclusions**: OCT findings correlated with visual impairment may help stratify disease severity at diagnosis. Prior corticosteroid exposure was associated with a lower yield of first-attempt vitrectomy, supporting steroid withdrawal before biopsy when feasible. These OCT features occurred together rather than independently, and are therefore best interpreted as a single imaging phenotype.

## 1. Introduction

Primary vitreoretinal lymphoma (PVRL) is a rare, aggressive subtype of extranodal diffuse large B-cell lymphoma. It affects the retina and vitreous and shares some similarities with primary central nervous system lymphoma. As its clinical presentation frequently overlaps with chronic posterior uveitis, PVRL is considered one of the classic ocular “masquerade syndromes,” and diagnosis is often delayed by months to years [1,2]. The broader diagnostic and therapeutic landscape of PVRL, including multimodal imaging, cytological and molecular confirmation, and current treatment strategies, has been reviewed in detail by our group in a clinical case published earlier in 2026 [3] and by others in various studies [4,5].

Optical coherence tomography (OCT) has steadily become one of the most informative non-invasive tools in suspected PVRL, with reported features including outer retinal hyperreflective infiltrates, nodular sub-retinal pigment epithelium (sub-RPE) deposits, and disruption of the outer retinal architecture [4,6,7]. However, most published series describe the frequency of individual OCT signs without quantifying their relationship to visual function, and the added value of combining multiple OCT features into a single severity measure has not been systematically explored. Similarly, while corticosteroid pretreatment is known to reduce the cellularity of vitreous specimens, its measurable effect on the diagnostic yield of vitrectomy in unselected cohorts remains sparsely documented [2,8].

Based on these findings, we also examine whether the individual OCT features analysed behave as independent markers or as components of a single co-occurring imaging phenotype, an issue that bears directly on how such features should be interpreted at the bedside.

## 2. Materials and Methods

### 2.1. Study Design and Setting

This retrospective, observational cohort study was conducted at the “Prof. Dr. Mircea Olteanu” Clinical Institute of Ophthalmological Emergencies and the Retina Clinic, both in Bucharest, Romania. Medical records of patients diagnosed with PVRL between January 2018 and December 2023 were reviewed.

### 2.2. Patients

Eligible patients were >18 years old, had a cytologically confirmed diagnosis of PVRL, and had complete clinical and imaging data available at the time of diagnosis. Patients with secondary intraocular lymphoma, other lymphoproliferative disorders with ocular involvement, cases lacking cytological confirmation, or incomplete documentation were excluded. Seventeen patients met the inclusion criteria.

### 2.3. Clinical and Imaging Assessment

For each patient, demographic characteristics, medical history (including prior corticosteroid exposure), presenting symptoms, best-corrected visual acuity (VA), biomicroscopy and fundus findings, and OCT features were recorded. Visual acuity was converted to logMAR units for statistical analysis; counting fingers and hand motion were assigned values of 1.9 and 2.3 logMAR, respectively, per standard convention. In bilateral cases, the eye with the worse VA was used for analysis.

Imaging was performed with spectral-domain OCT only. Fundus autofluorescence, fluorescein angiography and indocyanine green angiography were not systematically acquired and are not reported here; the imaging assessment in this study is therefore based on a single modality, and the term multimodal imaging is not used to describe it.

The OCT examinations analysed in this study are those performed at the time PVRL was first suspected and the diagnostic work-up initiated, not those obtained at the initial onset of vitritis, which in most patients predated referral to our centres and was documented elsewhere. Patients underwent repeated OCT imaging during subsequent treatment and follow-up; those longitudinal data are not analysed here, and the present analysis is cross-sectional, describing the imaging phenotype at the point of diagnostic suspicion.

The diagnosis was established by cytological examination of vitreous biopsy material, interpreted in correlation with clinical and imaging findings. Ancillary diagnostic techniques—immunocytochemistry, flow cytometry, intraocular cytokine measurement (IL-10/IL-6 ratio), and molecular testing for immunoglobulin heavy chain rearrangement or the MYD88 L265P mutation—were not available at the participating centres during the study period and were not performed in any of the 17 patients. Diagnosis therefore rested on cytology alone, and the implications of this for case ascertainment are addressed in the Limitations.

Both participating centres are dedicated ophthalmology units and do not perform neuroimaging or cerebrospinal fluid analysis on site. Patients were referred externally for neurological and haematological assessment, and the outcome was recorded in the ophthalmology notes as the presence or absence of central nervous system (CNS) lymphoma; this binary status was available for all 17 patients. The date on which CNS disease was established relative to the ocular diagnosis was not consistently recorded, so the sequence of ocular and CNS involvement could not be reconstructed.

### 2.4. Exploratory OCT Score

A five-item exploratory OCT score was constructed by summing the binary presence (0 = absent; 1 = present) of the following features: retinal infiltrates, sub-RPE lesions, outer retinal hyperreflectivity, outer retinal disorganization, and foveal involvement, yielding a total score from 0 to 5. This score was used exclusively to explore the relationship between cumulative structural burden and visual function; it has not been externally validated.

### 2.5. Statistical Analysis

Data were analyzed using IBM SPSS Statistics for Windows, version 26 (IBM Corp., Armonk, NY, USA). Normality was assessed with the Shapiro–Wilk test. Continuous variables are reported as mean ± standard deviation or median [interquartile range], as appropriate; categorical variables are reported as frequencies and percentages. Given the small sample size and non-normal distribution of most parameters, associations between continuous variables were assessed using Spearman’s rank correlation coefficient (ρ). Between-group comparisons of continuous variables used the Mann–Whitney U test; associations between categorical variables used Fisher’s exact test. All tests were two-tailed, with statistical significance set at *p* < 0.05.

### 2.6. Ethics

The study was conducted in accordance with the Declaration of Helsinki and applicable national data-protection legislation. The research protocol was approved by the Ethics Committee of the “Prof. Dr. Mircea Olteanu” Clinical Institute of Ophthalmological Emergencies, Bucharest, Romania (approval no. 1289/15.02.2026); patient data were anonymized prior to analysis.

### 2.7. Use of Generative AI and AI-Assisted Technologies

During the preparation of this manuscript, the authors used ChatGPT (GPT5, OpenAI, San Francisco, CA, USA) and Claude (Fable5, Anthropic, San Francisco, CA, USA) for language editing, improvement of clarity and structure, and preparation of the composite figure of representative fundus and OCT images. The tools were not used to generate or analyze patient data, statistical results, references, or scientific conclusions. No identifiable patient information was entered into either platform. The authors reviewed and verified all AI-assisted content and take full responsibility for the final manuscript.

## 3. Results

### 3.1. General Cohort Characteristics

Seventeen patients diagnosed with PVRL between 2018 and 2023 were included; 14 (82.4%) were evaluated at the Clinical Institute of Ophthalmological Emergencies and 3 (17.6%) at the Retina Clinic. Mean age at diagnosis was 66.1 ± 9.6 years (median 67, range 43–80), with a marked female predominance (13/17, 76.5%; F:M ≈ 3.3:1). Median symptom duration before diagnosis was 250 days [IQR 238–290]. Bilateral involvement was present in 76.5% of patients at diagnosis and reached 88.2% during follow-up, as two of the four initially unilateral cases developed contralateral disease (Table 1).

### 3.2. Presenting Symptoms and Visual Acuity

Decreased VA was the most common presenting complaint (15/17, 88.2%), followed by floaters (10/17, 58.8%); one patient presented with conjunctival hyperemia. Mean VA at diagnosis was 1.22 ± 0.70 logMAR (median 1.00 [IQR 0.82–1.90]). Neither age (ρ = −0.115, *p* = 0.661) nor symptom duration (ρ = 0.056, *p* = 0.832) correlated significantly with VA, suggesting that functional impairment is driven predominantly by the extent and location of intraocular infiltration rather than by patient age or disease chronicity.

Central nervous system lymphoma was documented in 10 of the 17 patients (58.8%); the remaining 7 (41.2%) had no recorded CNS involvement. Because the timing of the CNS diagnosis relative to the ocular presentation was not consistently recorded, these patients cannot be classified as primary ocular disease with subsequent CNS spread versus ocular involvement of established CNS lymphoma.

### 3.3. Clinical and OCT Findings

Vitritis was the most frequent objective finding (16/17, 94.1%). On OCT, retinal infiltrates, sub-RPE lesions, outer retinal hyperreflectivity, and outer retinal disorganization were each present in 13/17 patients (76.5%); foveal involvement was present in 10/17 (58.8%). Subretinal fluid was the least common feature (2/17, 11.8%) (Table 2).

### 3.4. Relationship Between OCT Findings and Visual Acuity

The exploratory OCT score correlated strongly with VA (ρ = 0.772, *p* < 0.001), indicating that a greater number of structural abnormalities was associated with worse visual function. On individual comparison, VA was significantly worse in patients with outer retinal hyperreflectivity (*p* = 0.018), sub-RPE lesions (*p* = 0.016), retinal infiltrates (*p* = 0.016), and outer retinal disorganization (*p* = 0.020). Foveal involvement (*p* = 0.463) and subretinal fluid (*p* = 0.406) were not significantly associated with VA.

The composite score was distributed as follows: one patient scored 0, no patient scored 1, three scored 2, two scored 3, five scored 4 and six scored 5 (median 4, range 0–5). Scores were concentrated at the upper end of the scale, with 11 of 17 patients (64.7%) scoring 4 or 5.

The features included in the score did not occur independently of one another. Each of the four non-foveal features was present in 13 of 17 patients (76.5%), but not in the same 13 patients in every instance: 10 patients (58.8%) had all four simultaneously and 12 (70.6%) had at least three. These features therefore behave largely as components of a single co-occurring imaging phenotype rather than as separable findings, and the four individual comparisons reported above should be regarded as largely overlapping expressions of the same underlying contrast rather than as four independent results. The composite score functions in practice as a graded indicator of how fully this phenotype is expressed, and its correlation with VA should be interpreted in that light.

### 3.5. Cytological Confirmation and Corticosteroid Exposure

Diagnosis was confirmed on the first vitreous biopsy in 14/17 patients (82.4%); the remaining three (17.6%) required a repeat procedure due to an initially inconclusive cytology. Repeat biopsy was not associated with OCT score (*p* = 0.295) or baseline VA (*p* = 0.124). Prior corticosteroid therapy, however, was significantly associated with an inconclusive first cytology (Fisher’s exact *p* = 0.015): three of five patients with prior corticosteroid exposure required repeat biopsy, versus none of the twelve without such exposure (Table 3).

### 3.6. Representative Imaging Findings

**Figure 1:**
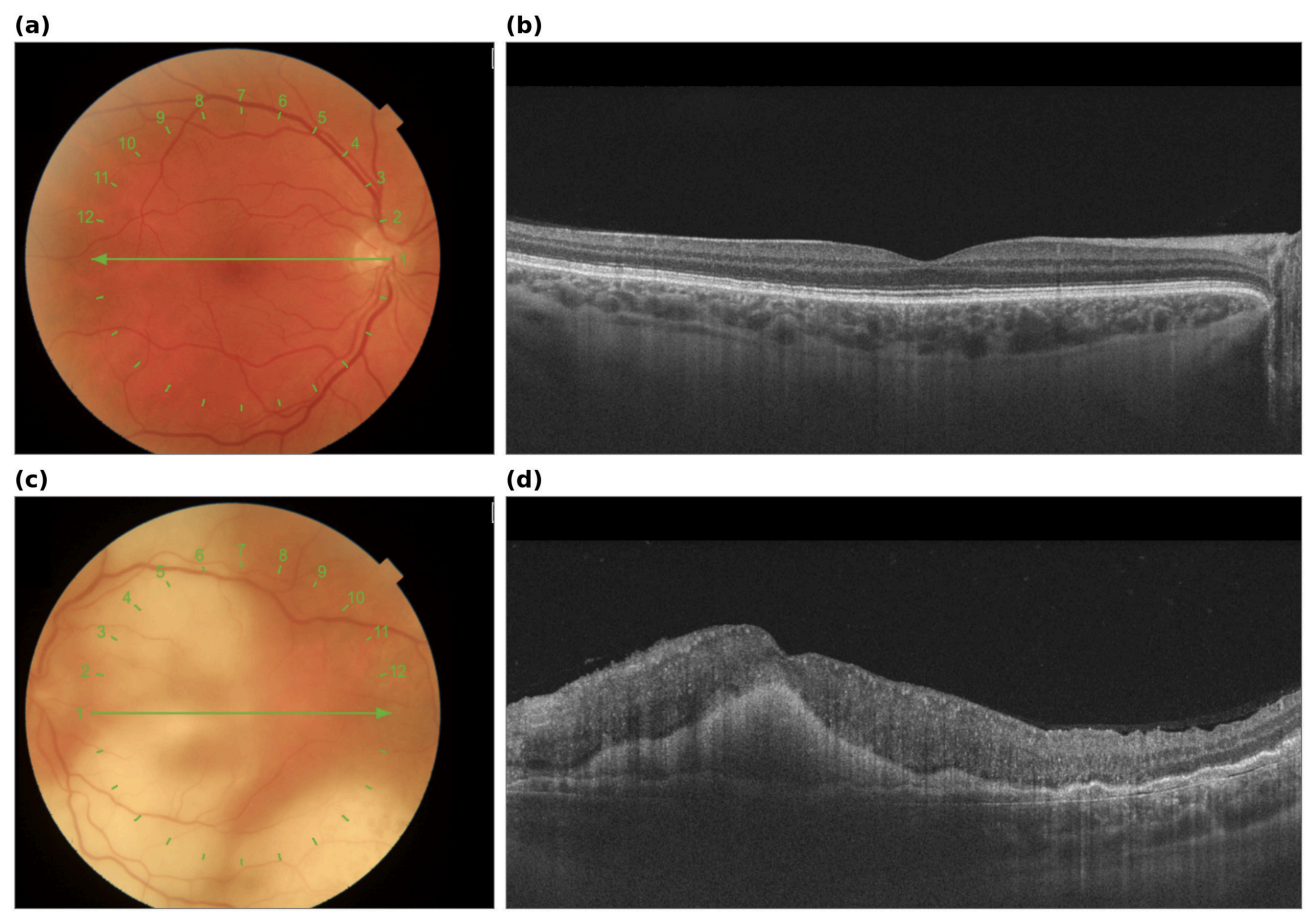
Representative imaging findings at diagnosis in a patient included in the cohort, showing bilateral but markedly asymmetric involvement. (**a**,**b**) Right eye: colour fundus photograph with preserved chorioretinal appearance, and the corresponding OCT B-scan showing relatively preserved retinal architecture with fine irregularity of the retinal pigment epithelium (RPE). (**c**,**d**) Left eye: colour fundus photograph showing diffuse creamy-yellow subretinal infiltration, and the corresponding OCT B-scan showing a dome-shaped elevation with hyperreflective material beneath the RPE and disorganization of the outer retinal layers. Both eyes were imaged on the same day. The green markers on the fundus photographs indicate the positions of the OCT scan lines. RPE, retinal pigment epithelium.

**Figure 2:**
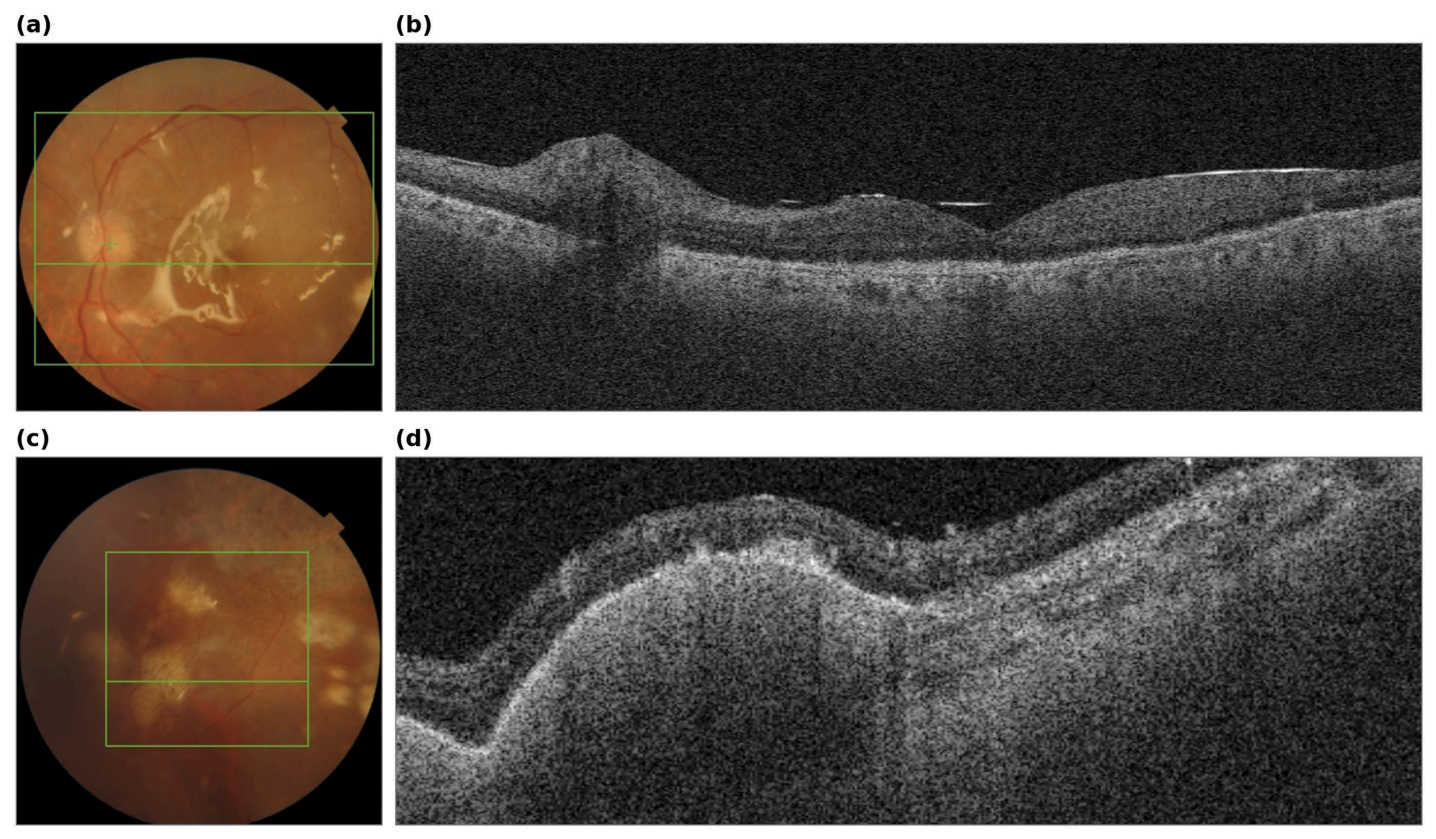
Colour fundus photographs and corresponding OCT B-scans from a second patient in the cohort. (**a**,**b**) Multiple discrete creamy-yellow subretinal infiltrates, with corresponding nodular elevations of the retinal pigment epithelium (RPE) and hyperreflective sub-RPE material on OCT. (**c**,**d**) Fellow eye with confluent subretinal infiltration and more extensive sub-RPE deposition with disorganization of the outer retinal layers. The green rectangles on the fundus photographs indicate the OCT scan areas. RPE, retinal pigment epithelium.

Representative colour fundus and OCT images from two patients in the cohort are shown in Figure 1 and Figure 2, illustrating the structural features included in the exploratory score and the bilateral but asymmetric pattern of involvement typical of this disease.

## 4. Discussion

Our study manages to quantify, in a well-characterized bi-centric cohort, the impact of OCT imaging in relation to visual function in assessing PVRL. Moreover, we have noted a measurable, statistically significant link between prior corticosteroid treatment and a lower diagnostic yield on the first vitrectomy. The demographic profile of our cohort—mean age 66.1 years, female predominance, and a high rate of bilateral involvement—is consistent with previously published series [3,9].

### 4.1. Cumulative Structural Burden on OCT and Visual Function

Furthermore, we showed that the correlation between cumulative structural involvement, resulting from our descriptive work in the form of an exploratory OCT score, and VA (ρ = 0.772, *p* < 0.001) best reflects functional impairment, whilst individual features can be associated with worse functional outcomes. Outer retinal hyperreflectivity, sub-RPE lesions, retinal infiltrates, and outer retinal disorganization—all features reflecting direct tumoral infiltration and photoreceptor architectural disruption—were each individually associated with worse VA, whereas foveal involvement and subretinal fluid were not. This is likely due to the limited number of affected eyes and possibly to the more variable functional impact of parafoveal versus subfoveal lesion location.

The individual OCT features observed in our cohort reproduce the imaging phenotype described in previous imaging series, in which outer retinal and sub-RPE hyperreflective deposits are recognized as the most characteristic signs of lymphomatous infiltration [6,10]. That these features tend to occur together has likewise been described previously and is not a novel observation; our data confirm it in an independent cohort and, more importantly, quantify its consequence. Because the features co-occur, they cannot be treated as independent contributors to visual loss, and the apparent significance of each individual comparison reflects a single underlying contrast between eyes with and without the phenotype.

What this permits is a more modest but clearer statement than the one we originally advanced. The cumulative score does not identify which feature matters most, because the features are not separable; it identifies whether the phenotype is present, and its strong correlation with VA (ρ = 0.772) indicates that the presence of this phenotype is associated with substantially worse visual function at diagnosis. The score was constructed post hoc, was not weighted, carries no diagnostic threshold, and given the bimodal distribution observed it should be regarded as an indicator of phenotype presence rather than as a graded measure of severity. It is a descriptive research instrument and not a clinical decision tool.

### 4.2. Corticosteroid Exposure and Diagnostic Yield

The cells encountered in PVRL are usually fragile diffuse large B-cell lymphoma cells with limited ex vivo viability and a high tendency toward spontaneous degeneration. Corticosteroids exert direct lympholytic and pro-apoptotic effects on these specific cells, thus temporarily reducing the amount of vitreous infiltration and subretinal infiltration. Therefore, if corticosteroids were administered less than 2 weeks before performing diagnostic vitrectomy, the biopsy specimens could still be influenced by the treatment and may be paucicellular, containing fewer viable malignant cells. This could impair morphological identification, immunophenotyping, and cytological interpretation, thereby increasing the risk of a false-negative or inconclusive result [2,5,8]. Most probably, this mechanism may explain why corticosteroid-pretreated patients in our cohort more frequently required repeat vitreous sampling. The current literature and international consensus recommendations therefore advise discontinuing systemic corticosteroids for at least 2 weeks before diagnostic vitrectomy, whenever clinically feasible [5,11]. Nevertheless, because our analysis was retrospective and included a small number of exposed patients, and because corticosteroid dose, route, duration, and withdrawal interval were not standardized, the observed association should not be interpreted as definitive evidence of causality. When corticosteroids cannot be safely withheld or when biopsy cannot be postponed, a negative cytological result should be interpreted cautiously and correlated with clinical findings, multimodal imaging, and complementary molecular or cytokine-based investigations; repeat biopsy should be considered when clinical suspicion remains high [2,5,11].

### 4.3. Diagnostic Delay

One of the most striking observations in our cohort was the length of the interval between symptom onset and diagnosis, with a median of 250 days [IQR 238–290]. This corresponds to more than eight months of untreated intraocular malignancy. Although unfortunate, this observation is consistent with the recognized tendency of PVRL to be diagnosed late [1,3]. More precisely, the mechanism is well illustrated by our own data: nearly all patients presented with vitritis, and five had already received corticosteroids before referral. A partial, transient symptomatic improvement under corticosteroid therapy is exactly the response pattern that reinforces an inflammatory working diagnosis and postpones biopsy, while simultaneously reducing the cellular yield of that biopsy once it is finally performed. Thus, corticosteroid responsiveness in this context is doubly misleading. An incomplete or short-lived response to steroids in an older patient with vitritis should be regarded as an argument for, rather than against, proceeding to diagnostic vitrectomy.

### 4.4. Bilaterality and Demographic Profile

Bilateral involvement was present in 76.5% of our patients at diagnosis and rose to 88.2% during follow-up, since two of the four initially unilateral cases developed disease in the fellow eye. Bilateral or asymmetric involvement is a recognized feature of PVRL [3,12], but the conversion observed here has a direct practical consequence: an apparently uninvolved fellow eye in a patient with confirmed PVRL should not be considered disease-free, and warrants structured imaging surveillance rather than clinical examination alone. Subtle outer retinal changes may precede symptomatic involvement, and OCT is well suited to detecting them at a stage when visual acuity is still preserved.

The female predominance in our cohort (76.5%; F:M ≈ 3.3:1) was more pronounced than the modest female excess usually described in larger series [12,13], and most likely reflects the small size of a single-country cohort rather than a genuine epidemiological difference. Notably, neither age nor symptom duration correlated with visual acuity at presentation. The absence of a relationship with symptom duration is clinically relevant because it argues against using reported disease duration as a surrogate for severity; symptom duration is subject to recall bias and to the variable point at which patients seek care, whereas the anatomical extent of infiltration, as captured on OCT, appears to be the stronger determinant of visual function.

### 4.5. Diagnostic Resources and Applicability

Another important contextual aspect of this cohort is that none of the ancillary techniques commonly used to support a diagnosis of PVRL—immunocytochemistry, flow cytometry, intraocular cytokine measurement (IL-10/IL-6 ratio), and molecular testing for immunoglobulin heavy chain rearrangement or the MYD88 L265P mutation—formed part of the routine diagnostic work-up at the participating centres during the study period. All diagnoses were therefore established through the combination of clinical assessment, OCT imaging, and conventional cytology alone. Although this limits the depth of biological characterization, it also means that the diagnostic pathway described here reflects the conditions under which PVRL is diagnosed in many centers worldwide, where consensus-recommended ancillary testing [11] is not accessible. In such settings, structured imaging assessment and rigorous attention to the conditions of biopsy—particularly corticosteroid withdrawal—carry proportionally greater weight, since there is no molecular test available to compensate for a paucicellular or inconclusive specimen.

### 4.6. Strengths and Limitations

The strengths of this study include the inclusion of consecutive patients from two referral centers, cytological confirmation of the diagnosis in every case, and the availability of complete clinical and OCT data at diagnosis for all patients, which allowed a uniform assessment of imaging features across the cohort.

The study is further limited by its retrospective design and small sample size, both reflecting the rarity of PVRL. The analyses were exploratory and not adjusted for multiple comparisons, so the individual *p*-values should be interpreted as hypothesis-generating rather than confirmatory; this applies with particular force to the four comparisons of individual OCT features, which, as shown above, are not statistically independent of one another. The corticosteroid analysis rests on five exposed patients, which precludes any estimate of effect size and any adjustment for confounding, and the dose, route, duration and withdrawal interval of prior corticosteroid therapy were not standardized.

The imaging assessment was based on spectral-domain OCT alone. Fundus autofluorescence and angiographic studies, which contribute to the recognition of PVRL in other series, were not systematically available, so the imaging phenotype described here is necessarily partial. Finally, the exploratory OCT score requires validation in an independent, prospective, multicenter cohort before any clinical use. An earlier version of this manuscript included a proposed diagnostic workflow; we have removed it, since a cohort of 17 patients cannot support a diagnostic algorithm, and the elements of such a workflow that our data do support—withholding corticosteroids before biopsy, and repeating the biopsy when suspicion persists—are stated directly in the text.

### 4.7. Future Directions

Prospective validation of the exploratory OCT score in a larger, multicenter cohort would clarify whether the co-occurring imaging phenotype retains its relationship with visual function and whether it carries prognostic value for treatment response. Incorporating molecular and cytokine testing as these become more widely available would allow the imaging phenotype to be correlated with biological markers of disease activity, and would help determine whether specific OCT patterns identify patients in whom cytology alone is likely to be insufficient. Given the rarity of the disease, collaborative registries offer the most realistic route to cohorts of adequate size [12]. Finally, automated analysis of OCT volumes may eventually allow the qualitative descriptors used here to be replaced by reproducible quantitative measures of infiltration.

## 5. Conclusions

In this retrospective bi-centric cohort, the OCT features analysed occurred together as a single imaging phenotype rather than independently, and the presence of this phenotype correlated strongly with worse visual acuity at diagnosis. Prior corticosteroid exposure was associated with a lower diagnostic yield on first vitrectomy. These findings support systematic OCT assessment in suspected PVRL and reinforce the recommendation to withhold corticosteroids before diagnostic biopsy whenever this is clinically feasible. Given the small sample size and the exploratory nature of the analyses, these observations should be regarded as hypothesis-generating; prospective, multicenter studies incorporating molecular biomarkers are needed to validate them.

## Figures and Tables

**Table 1:** Baseline demographic and clinical characteristics of the study cohort.

Characteristic	Value (*n* = 17)	Additional Summary
Age at diagnosis, years	66.1 ± 9.6	mean ± SD; median 67 (range 43–80)
Sex, female	13 (76.5%)	F:M ratio ≈ 3.3:1
Symptom duration before diagnosis, days	267.1 ± 73.2	median 250 [IQR 238–290]
Bilateral involvement at diagnosis	13 (76.5%)	
Bilateralization during follow-up	2 (11.8%)	
Bilateral involvement, overall	15 (88.2%)	
Decreased visual acuity at presentation	15 (88.2%)	most common presenting symptom
Floaters at presentation	10 (58.8%)	

Abbreviations: SD, standard deviation; IQR, interquartile range; F:M, female-to-male ratio. Percentages use *n* = 17 as the denominator.

**Table 2:** Frequency of clinical and OCT findings and their association with visual acuity.

Finding	Frequency	Direction of Association with VA (logMAR) *	*p*-Value
Vitritis	16 (94.1%)	not tested	–
Retinal infiltrates	13 (76.5%)	higher in presence	0.016
Sub-RPE lesions	13 (76.5%)	higher in presence	0.016
Outer retinal hyperreflectivity	13 (76.5%)	higher in presence	0.018
Outer retinal disorganization	13 (76.5%)	higher in presence	0.020
Foveal involvement	10 (58.8%)	no significant difference	0.463
Subretinal fluid	2 (11.8%)	no significant difference	0.406

* Higher logMAR values indicate worse visual acuity. *p*-values are from two-sided Mann–Whitney U comparisons of patients with versus without each finding; vitritis was not tested. Abbreviations: VA, visual acuity; logMAR, logarithm of the minimum angle of resolution; OCT, optical coherence tomography; RPE, retinal pigment epithelium.

**Table 3:** Association between prior corticosteroid exposure and first-biopsy diagnostic yield.

Prior Corticosteroid Exposure	Repeat Biopsy Required	Single Biopsy Diagnostic
Prior corticosteroid therapy (n = 5)	3	2
No prior corticosteroid therapy (n = 12)	0	12

Two-sided Fisher’s exact test: *p* = 0.015.

## Data Availability

The data presented in this study are available upon request from the corresponding author, subject to institutional data-protection restrictions.
